# The complete plastid genome of *Vincetoxicum junzifengense* B.J. Ye and S.P. Chen (Apocynaceae)

**DOI:** 10.1080/23802359.2022.2116955

**Published:** 2022-09-15

**Authors:** Bao-Jian Ye, Xia-Bing Shen, Yu-Wei Wu, Xue-Fen Cao, Xing-Wen Zhou

**Affiliations:** a College of Architecture and Urban Planning, Fujian Universtiy of Technology, Fuzhou, China; b College of Landscape, Fujian Agriculture and Forestry University, Fuzhou, China; c Junzifeng National Nature Reserve, Sanming, China

**Keywords:** Apocynaceae, Asclepiadoideae, chloroplast genome, phylogenetic analysis

## Abstract

*Vincetoxicum junzifengense* B.J. Ye and S.P. Chen 2022 is a newly described species which belongs to the genus *Vincetoxicum* in the family Apocynaceae. The complete plastid genome of *Vincetoxicum junzifengense* B.J. Ye and S.P. Chen 2022 was determined and analyzed in this study. The total chloroplast genome was 159,666 bp in length, consisting of a large single-copy region of 90,565 bp, a small single-copy region of 19,691 bp, and two inverted repeat regions of 24,705 bp. The genome contained 131 genes, including 85 protein-coding genes, 37 transfer RNA genes, and eight ribosomal RNA genes. Phylogenetic analysis indicated that *V. junzifengense* is sister to *V*. *versicolor.*

*Vincetoxicum*, which consists of approximately 200 species, is one of the most species-rich genera of Apocynaceae (Shah et al. 2021). They are distributed in Asia, Africa, Arabia, Australia, and Eurasia (Douglass et al. 2009) and China is one of the most diverse centers, with 71 species of the *Vincetoxicum* genus distributed in China (Jiang et al. 2018). *Vincetoxicum junzifengense* B.J. Ye and S.P. Chen 2022 is a newly described species and characterized by its fascicled and slender roots, narrowly lanceolate leaf blade, raceme-like inflorescences, amaranth corolla with triangular lobes, cupular corona with triangular and dentate lobes, pentagon and slightly elevated stigma, two ovoid pollinia per pollinarium, single lanceolate and glabrous follicles. This species was only found at one site in the Junzifeng National Nature Reserve, West Fujian, China, and grows in the subtropical evergreen broad-leaved forest, moist and loose soil. From morphological aspects, *V. junzifengense* is similar to *V. versicolor* and *V. stauntonii* and our phylogenetic analysis supported that *V. junzifengense* is a sister to *V*. *versicolor* (Ye et al. 2022). In this study, the complete plastid genome of this species was assembled, annotated and analyzed.

The samples were collected from Mingxi district, Fujian, China (25°35′N, 116°55′E) and the voucher specimen deposited at Herbarium of Fujian Agriculture and Forestry University (http://lxy.fafu.edu.cn/, contact person: Chen Shipin; email: fjcsp@126.com) under the voucher code Baojian Ye WSD9808. Total genomic DNA was extracted from fresh leaves using a modified CTAB method of Doyle (Doyle 1987) and sequenced by the BGISEQ-500 platform. The GetOrganelle v1.7.5 (Jin et al. 2020) was used to filter the raw sequence reads to get the high-quality plastid like reads. And then the target-associated reads produced by the former step to get the final FASTA files were assembled using SPAdes within the same pipeline (Bankevich et al. 2012). With the chloroplast genome of *Vincetoxicum versicolor* (GenBank accession NC_052877) as the reference sequences, the assembled plastid genome was annotated using the Geneious R11.15 (Kearse et al. 2012).

The total plastid genome of *V. junzifengense* (GenBank accession OM995819) was 159,666 bp in length, consisting of a large single-copy (LSC) region of 90,565 bp, a small single-copy (SSC) region of 19,691 bp, and two inverted repeat regions (IRA and IRB) of 24,705 bp. The GC content of the chloroplast genome was 37.80%, while the corresponding values of the LSC, SSC, and IR regions are 56.72%, 12.33%, and 30.95%, respectively. The complete chloroplast genome contains 131 genes, including 85 protein-coding genes, 37 transfer RNA (tRNA) genes, and eight ribosomal RNA (rRNA) genes.

To detect the phylogenetic relationship of *V. junzifengense* with other Apocynaceae members, additional 27 representative species of Apocynaceae were downloaded from NCBI, with two species of Loganiaceae and one species of Gentianaceae as outgroups. Data matrices were aligned by MAFFT v7 (Katoh and Standley 2013) plugin in the software Phylosuite v1.2.2 (Zhang et al. 2020). After nucleotide sequence alignment, the phylogenetic analysis was performed based on the complete plastid genomes using the maximum likelihood (ML), which was conducted on the website CIPRES Science Gateway with RAxML-HPC2 on XSEDE 8.2.10 (Miller et al. 2010), and the phylogenetic tree constructed by RAxML (Stamatakis 2014) with 1,000 bootstrap replicates (Minh et al. 2013; Chernomor et al. 2016). Our molecular analysis indicates that *V. junzifengense* was sister to *V*. *versicolor* with strongly support (BP = 100) (Figure 1).

**Figure 1.**
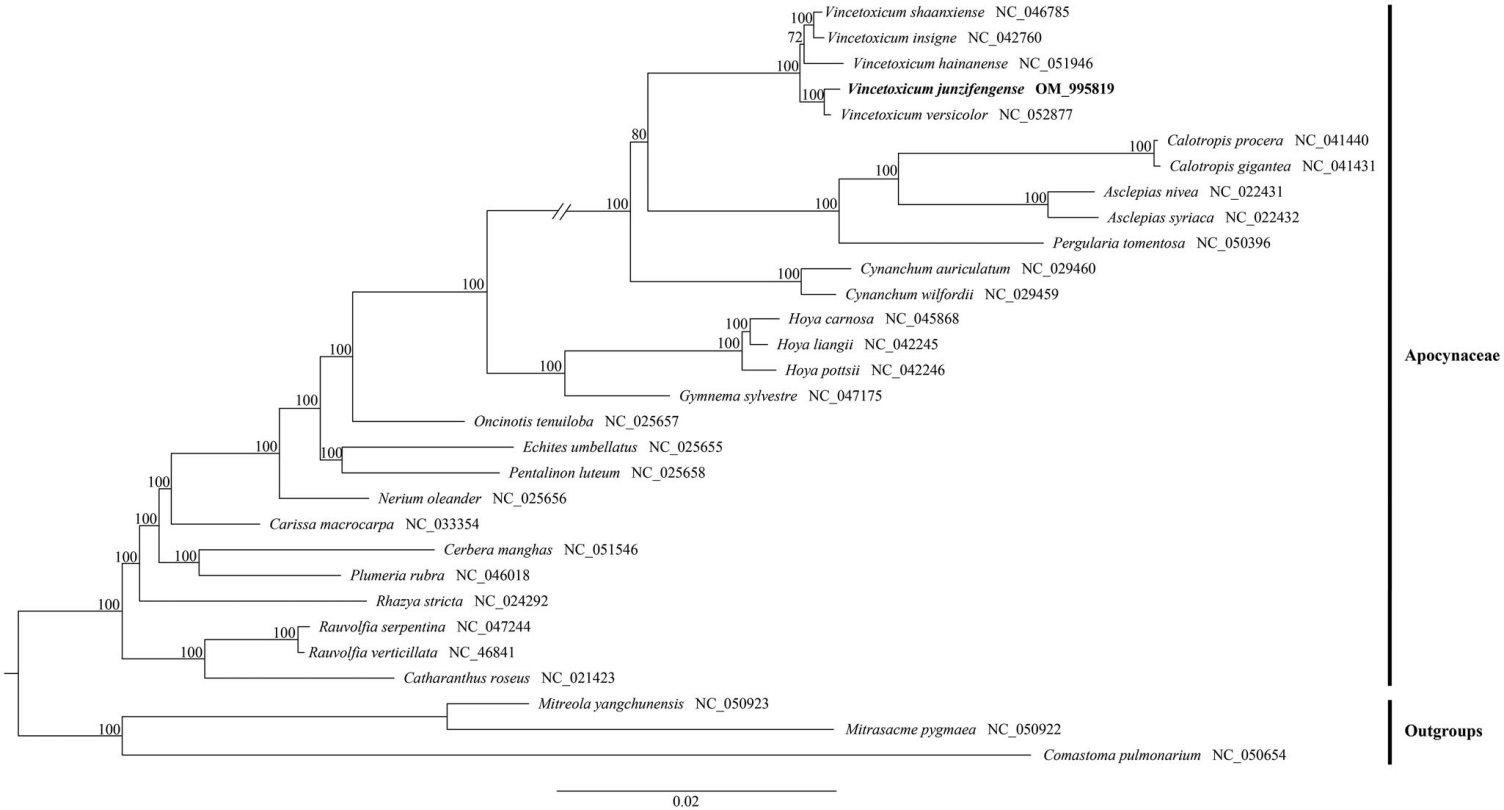
The maximum-likelihood (ML) tree based on the 27 representative plastid genome sequences of Apocynaceae and two outgroup species of Loganiaceae and one outgroup species of Gentianaceae. The bootstrap value near each node.

## Data Availability

The genome sequence data that support the findings of this study are openly available in GenBank of NCBI at (https://www.ncbi.nlm.nih.gov/) under the accession no. OM995819.1. The associated BioProject, Bio Sample and SRA, numbers are PRJNA845946, SAMN28865336, and SRR19543586 respectively.
